# Damage to the medial motor system in stroke patients with motor neglect

**DOI:** 10.3389/fnhum.2014.00408

**Published:** 2014-06-11

**Authors:** Raffaella Migliaccio, Florence Bouhali, Federica Rastelli, Sophie Ferrieux, Celine Arbizu, Stephane Vincent, Pascale Pradat-Diehl, Paolo Bartolomeo

**Affiliations:** ^1^Inserm UMR-S 1127, UPMC-Paris 6, CNRS UMR 7225, Brain and Spine Institute, Groupe Hospitalier Pitié-SalpêtrièreParis, France; ^2^Fédération de Neurologie, IM2A, AP-HP, Groupe Hospitalier Pitié-SalpêtrièreParis, France; ^3^Department of Psychology, Catholic UniversityMilan, Italy; ^4^AP-HP, Service de Médecine Physique et Réadaptation, Groupe Hospitalier Pitié-SalpêtrièreParis, France; ^5^ER 06, UPMC, Service de MPR, Groupe Hospitalier Pitié-SalpêtrièreParis, France

**Keywords:** motor neglect, visual neglect, medial motor system, cingulum bundle, parieto-frontal network

## Abstract

**Background and objectives:** Motor neglect (MN) is a clinically important condition whereby patients with unilateral brain lesions fail to move their contralateral limbs, despite normal muscle strength, reflexes, and sensation. MN has been associated with various lesion sites, including the parietal and frontal cortex, the internal capsule, the lenticulostriate nuclei, and the thalamus. In the present study, we explored the hypothesis that MN depends on a dysfunction of the medial motor system by performing a detailed anatomical analysis in four patients with MN.

**Methods:** Ten patients participated in the study: four with MN, four with left visual neglect but without MN, and three patients with left hemiplegia without MN. We used specific scales for clinical and neuropsychological assessment. We drew the lesion borders directly onto the original brain images of each patient, and plotted the lesions on anatomical atlases for gray and white matter.

**Results:** Lesion locations were highly heterogeneous in our MN patients, and included frontal and parietal sites, basal ganglia, and white matter. The only consistently damaged structure across all MN patients was the cingulum bundle, a major pathway of the medial motor system important for motor initiative, and a key connection with limbic structures crucial for motivational aspects of actions. Three MN patients with additional damage to lateral fronto-parietal networks had also signs of contralesional visual neglect. The cingulum bundle was intact in all the control patients with visual neglect or hemiplegia.

**Conclusions:** Cingulum damage may induce MN through unilateral dysfunction of the medial motor system. Additional lateral fronto-parietal dysfunction can result in the association with visual neglect.

## Introduction

Patients with unilateral brain damage may display underutilization of contralesional limbs that cannot be explained by primary sensori-motor deficits (Punt et al., 2013; Sampanis and Riddoch, 2013). This condition, known as motor neglect (MN) (Laplane and Degos, 1983), entails important clinical problems, because these patients may behave as if they were hemiplegic. Critchley (1953) listed MN among the consequences of parietal damage. In its pure form (without hemiparesis), MN is a relatively rare disorder in chronic stroke patients. For example, Laplane and Degos (1983) collected 20 patients over more than 10 years. In their series, MN was not always associated with “sensory” (i.e., visuospatial) neglect. In other studies, signs of MN occurred in 12–33% of acute stroke patients (Buxbaum et al., 2004; Siekierka-Kleiser et al., 2006). The frequency decreased to 8% in chronic patients (Buxbaum et al., 2004). In one study (Classen et al., 1997), 10 out of 16 patients with MN improved during the first 2 weeks (review in Saevarsson, 2013).

Clinically, underutilization is often evident in bimanual tasks (e.g., opening a bottle), as in “motor extinction” (Valenstein and Heilman, 1981), or as decreased automatic gesturing during walking or talking. Strong prompting can usually induce movements of the neglected limbs.

Because movements in response to a strong prompting (such as in the testing set) are typically preserved in MN, this condition is particularly difficult to study in an objective manner. Experimental studies exploring the MN have proposed methods to investigate in details some aspects of the syndrome, such as motor extinction or lateralized inhibitory deficits. In a case report, Punt et al. (2005) compared unimanual and bimanual reaching movements (to grasp movements toward one or two objects) and showed that motor extinction was influenced by visual grouping between stimuli. Coulthard et al. (2008) studied seven patients affected by MN, and they showed that when patients with left MN planned to move their left hand, irrelevant right limb motor programmes intruded, causing delay.

For the present study, we collected a list of clinical observations able to detect the presence and severity of MN. Even if MN is independent of elementary motor deficits, it may frequently co-exist with them. In order to obtain reliable information on the anatomy, we recruited only chronic patients with preserved strength at the time of inclusion.

MN has been associated with various lesion sites, including the parietal and frontal cortices, as well as the internal capsule, lenticulostriate nuclei, and thalamus (Valenstein and Heilman, 1981; Laplane and Degos, 1983; Bogousslavsky et al., 1986; De La Sayette et al., 1992; Manabe et al., 1999; Coulthard et al., 2008). Most MN patients (74% in Siekierka-Kleiser et al., 2006) have a right hemisphere lesion, but MN can also occur after left hemisphere damage (Siekierka-Kleiser et al., 2006).

Recent models of initiation of motor activity (Haggard, 2008) postulate the existence of two main fronto-parietal motor systems. A lateral fronto-parietal network, connected by branches of the superior longitudinal fasciculus, is important for acts performed in response to environmental stimuli, and is thus related to spatial attention; a medial fronto-parietal network, connected by the cingulum bundle (Catani and Thiebaut de Schotten, 2012), would instead be necessary for spontaneous initiation of movement.

The main aim of the present multiple single-case study is to perform a detailed anatomical analysis of MN patients brain lesions. We also explored the relationships between MN, visual neglect and hemiplegia, examining additional control patients with left visual neglect (*N* = 4), or with left hemiplegia (*N* = 3).

Anatomo-functional predictions stemming from the proposed lateral/medial dichotomy are that neural damage should mainly involve the medial network in patients with MN, and the lateral network in patients with visual neglect. Both networks might instead be relatively preserved in patients with hemiplegia in the absence of cognitive deficits.

## Materials and methods

We had originally explored seven patients with unilateral hemispheric lesions and signs of MN, over a year.

Three patients were subsequently excluded from analysis because of complete recovery from MN (two cases, for whom CT or MRI was not obtained), or of the concomitant presence of severe executive deficits (one case). This patient was excluded because of severe frontal syndrome, which precluded a correct MN exploration. The four recruited MN patients were three men and one woman (mean age ± SD, 59 ± 8; Table 1). All the patients included were in the chronic post-stroke phase (time after stroke from 3 to 11 months).

**Table 1:** **Demographics and clinical characteristics of Motor Neglect patients**.

**Patient**	**Age**	**Gender**	**Manual preference**	**Time after stroke (months)**	**Lesion side**	**Etiology**	**MN (MN observation/Tea task scores)**	**Visuospatial neglect**	**Gray matter lesion site**	**White matter lesion site**	**Lesion volume (cm^3^)**
1	47	M	R	11	R	Ischemic	5/5	Yes, left-sided	None	Cingulum, SLF	7.39
2	53	F	R	3	R	Hemorrhagic	7/1	No	SMA, pre-SMA, superior and middle frontal gyrus, anterior cingulum, caudate nucleus, thalamus	Anterior cingulum	76.2
3	64	M	R	4	L	Ischemic	16/27	Yes, right-sided	Post-central gyrus, superior and inferior parietal gyrus, precuneus, superior occipital gyrus	Posterior cingulum, SLF	42.2
4	61	M	L	3	L	Hemorrhagic	5/0	Yes, right-sided	Putamen	Cingulum, SLF?	3.9

*L, left; R, right; SLF, superior longitudinal fasciculus; MN, motor neglect; SMA, supplementary motor area*.

*Scores for MN were calculated from the assessment adopted for this study*.

*For the MN observation scale, the score ranged from 0 (no MN) to 20 (extremely severe MN). For the evaluation of tea task, we used scores weighted differently, depending on whether the arm affected by MN did or did not correspond to the dominant arm (for example, right-handed patient with left MN vs. right-handed patient with right MN, see also the battery in supplemental material 1). The higher the score, the more severe the MN. For example, gripping the handle of the cup to lift it is usually performed with the dominant hand; if a patient did not take the cup with his/her dominant hand (affected by MN), then a higher MN score was attributed*.

Following previous studies (Coulthard et al., 2008), we developed a clinical scale, as a simple bedside test to assess the presence and severity of MN. Our scale is mainly based on signs and symptoms listed in the seminal paper by Laplane and Degos (Laplane and Degos, 1983). In the present study, we propose two levels of observation (by the examiners R.M. and F. B. and by the paramedical staff), and an ecological task (see below). However, it should be noted that abnormal performance on this scale could also result from other disorders, including unilateral Parkinson's disease. Thus, performance on this scale has to be interpreted in the appropriate clinical context.

### MN assessment

Our clinical scale (see Table S2) included the following items: limbs positioning, symmetry of the posture, presence/absence of a “placing reaction,” hand gesturing during speaking, arm swing during walking, underutilization, hypometria, performance in bimanual tasks, and ability to catch an object. Scores ranged from 0 (no MN) to 20 (extremely severe MN). A further section with similar items was the object of an interview with paramedical staff. MN is by definition less apparent under explicit instructions; patients thus also performed a video-recorded ecological task (tea preparation). Their gestures and hand use were analyzed and scores were given to four phases: 1-use of teapot, 2-use of tea bag, 3-sugar adding, 4-cup grasping (Table S2). For the evaluation of the tea task, we used scores differently weighted, depending on whether the arm affected by MN was or not the dominant arm. For example, gripping the handle of the cup to lift it is usually performed with the dominant hand; if a patient did not take the cup with his/her dominant hand (affected by MN), then a higher MN score was attributed.

### Additional evaluations included

(1) *Muscle strength*. Neurological evaluation was performed first against gravity and then against full resistance for upper and lower extremities, and comparing both sides; scoring from 0 to 3 (0 = No hemiplegia; 3 = Complete hemiplegia) (see Garbarini et al., 2013). (2) *Visuo-spatial neglect*. Tests usually available in clinical practice, from the GEREN battery (Azouvi et al., 2002) (e.g., bells test; target cancellation; line bisection; clock or landscape drawing test; reading test), were used. (3) *Personal neglect*. A composite score was obtained from three tests (“eyeglasses—razor—comb” test Committeri et al., 2007, Bisach test Bisiach et al., 1986, and Fluff test Cocchini et al., 2001). Scores ranged from 0 (no personal neglect) to 10 (severe personal neglect).

### Clinical data

Here we present the clinical history of each patient, together with the assessment of muscle strength, visuo-spatial and personal neglect, as well as the lesion size.

*Patient 1* was a 47-year-old, right-handed man. He was tested 11 months after a watershed ischemia as consequence of right internal carotid artery dissection. At onset, he had severe left sided hemiplegia, which had significantly improved by the time. At the time of observation, the muscle strength was normal (hemiplegia score = 0). At testing, the patient had signs of left MN. In addition he showed mild executive deficits, in particular of impaired spatial working memory. Patient 1 also showed signs of mild left visual neglect on the clock-drawing test (omission of left-sided digits) and on the writing test (obtained score, 9.5 cm; normal < 6.7 cm). There was no evidence of personal neglect. The lesion size was 7.39 cm^3^.

*Patient 2* was a 53-year-old right-handed woman. Three months before evaluation, she underwent neurosurgery for the rupture of an artero-venous malformation in the right frontal lobe. At the time of our observation, in addition to signs of left MN, the patient also showed moderate executive deficits, mild memory disorders with some confabulations, and impaired recognition of negative facial expressions, such as fear. There were no elementary motor deficit (hemiplegia score = 0) or signs of visuo-spatial or personal neglect. The following visuo-spatial tests were performed: bells cancellation test (left/right hits, 13/15); 20-cm line bisection (mean leftward deviation, 4 mm; normal, less than 7.5 mm); overlapping figure identification, landscape drawing and reading test (no omissions). The personal neglect score was 0.18/10. The lesion size was 76.2 cm^3^.

*Patient 3* was a 64-year-old, right-handed man. Four months before evaluation, after two transient ischemic episodes presenting with right arm numbness, he had an ischemic stroke in the left hemisphere, with subsequent haemorrhagic transformation. At the time of hospitalization, patient 3 showed right-side MN and signs of mild right visual neglect. On line bisection, he deviated leftwards by 11% (normal, < 7.5%). He omitted right-sided digits on clock drawing test. There was also right tactile extinction in the context of mild right hypoesthesia, apraxia of speech and acalculia. A few days later, apraxia of speech and visual neglect had improved, and hemi-hypoestesia had completely regressed. There were no hemiparesis/hemiplegia (hemiplegia score = 0) or personal neglect (score = 0.84/10). The lesion was 42.2 cm^3^ in volume.

*Patient 4* was a 61-year-old, left-handed man. Three months before evaluation he had a left putaminal hematoma with subarachnoid hemorrhage, presenting with right side MN, personal and visual neglect, anomia, memory deficits, anosodiaphoria, and right hemiparesis. At the time of evaluation, the patient still had signs of MN. There were right-sided extinctions on double simultaneous visual stimulation and signs of mild right-sided visual neglect on target cancellation (17 right omissions out of 22 targets). Patient 4 had no hemiparesis/hemiplegia (score = 0), or personal neglect (score = 0/10). The lesion size was 3.9 cm^3^.

### Anatomical study

The MR protocol was carried out with a 3-Tesla whole-body system (Siemens, Erlangen, Germany) at the Center for Neuroimaging Research (CENIR), Brain and Spine Institute (ICM) in Paris. Lesion analysis was performed by an expert neurologist (RM) and a master student (FB) trained to read brain scans. Lesion extent was determined for each patient by selecting those brain scans that showed the greatest extent of damage (hypointense lesions on T1-weighted images). The lesion borders were directly drawn onto the original native 3D MRI, using MRIcro software (Rorden and Brett, 2000, www.mricro.com) and a graphic tablet (WACOM Intuos A6, Vancouver, Washington, USA). Afterwards, in order to plot all the patients' lesions in the same standard space, the 3D brain scans and lesion volumes were normalized to the standard Montreal Neurological Institute (MNI) brain template in Statistical Parametric Mapping 8 (http://www.fil.ion.ucl.ac.uk.gate2.inist.fr/spm) running under Matlab 7.13 (http://www.mathworks.com). In particular, to reduce lesion-induced registration errors, spatial normalization was performed using a mask that excluded the damaged areas of the brains, thereby preventing these areas from biasing the transformation. After normalization, brain lesions were segmented and their borders redefined in the normalized brain. MRIcron software (Rorden et al., 2007) (http://www.mccauslandcenter.sc.edu/mricro/mricron/) was used to measure the extent of the lesion. For patient 2, who could not undergo MRI, because of the presence of surgical metallic clips, the lesion was delineated using a similar procedure. Lesions were first drawn from high-resolution CT scans and then manually transposed onto the standard MNI brain template (for the methodology see also Bourgeois et al., 2012; Charras et al., 2012; Rastelli et al., 2013).

We used anatomical atlases, both traditional (Duvernoy atlas) and electronic (Tzourio-Mazoyer et al., 2002), to assess gray and white matter involvement. In particular, the involvement of white-matter bundles was assessed by cross-referencing neuroanatomical and previous DT MRI tractography works. A composite map, derived from the atlas proposed by Thiebaut de Schotten et al. (2011b) based on the DTI study of 40 normal subjects, was used. This atlas has been created to assess the relationship of focal lesions with nearby tracts and to easily improve clinico-anatomical correlation. In the reference article, the authors demonstrated that the atlas correctly identified the lesioned tracts (Thiebaut de Schotten et al., 2011b). To test for possible differences in the involvement of the two motor systems (lateral vs. medial) in patients' profiles, two main long-range white matter bundles were explored, the superior longitudinal fasciculus and the cingulum.

In order to further specify the lesional correlates of MN, we recruited additional patients with left visual neglect and patients with hemiplegia (without MN). The inclusion criteria were unilateral vascular damage with impaired performance on at least two tests of a systematic visual neglect battery of paper and pencil tests (Azouvi et al., 2002) for patients with signs of left visual neglect, and no deficit on any of the tests for patients without visual neglect. Four visual neglect (VN) patients (mean age 58.7 years, range 57–62) and three patients with hemiplegia (P) without signs of visual neglect (mean age 57 years, range 52–65) fulfilled the criteria and participated in the study. The mean time of testing for the included patients was 4.6 months since stroke onset (SD, 1.7 months).

For statistical analysis, we used MRIcron and non-parametric mapping, which is part of the MRIcron software package (Rorden et al., 2007). We contrasted MN patients and control groups (VN and H, both without MN) by using non-parametric voxel-based lesion symptom mapping (VLSM) analysis (Bates et al., 2003), and plotting all the lesions on the same (right) hemisphere. We used the Liebermeister test, which is a non-parametric implementation test of a two-group comparison on a binary variable (for details see also Heydrich and Blanke, 2013). Only voxels damaged in at least 20% of individuals were considered. We corrected the results for multiple comparisons using false discovery rate (FDR).

Approval was received from a local ethical standards committee on human experimentation and written informed consent was obtained from all patients prior to study enrolment.

## Results

**Figure 1:**
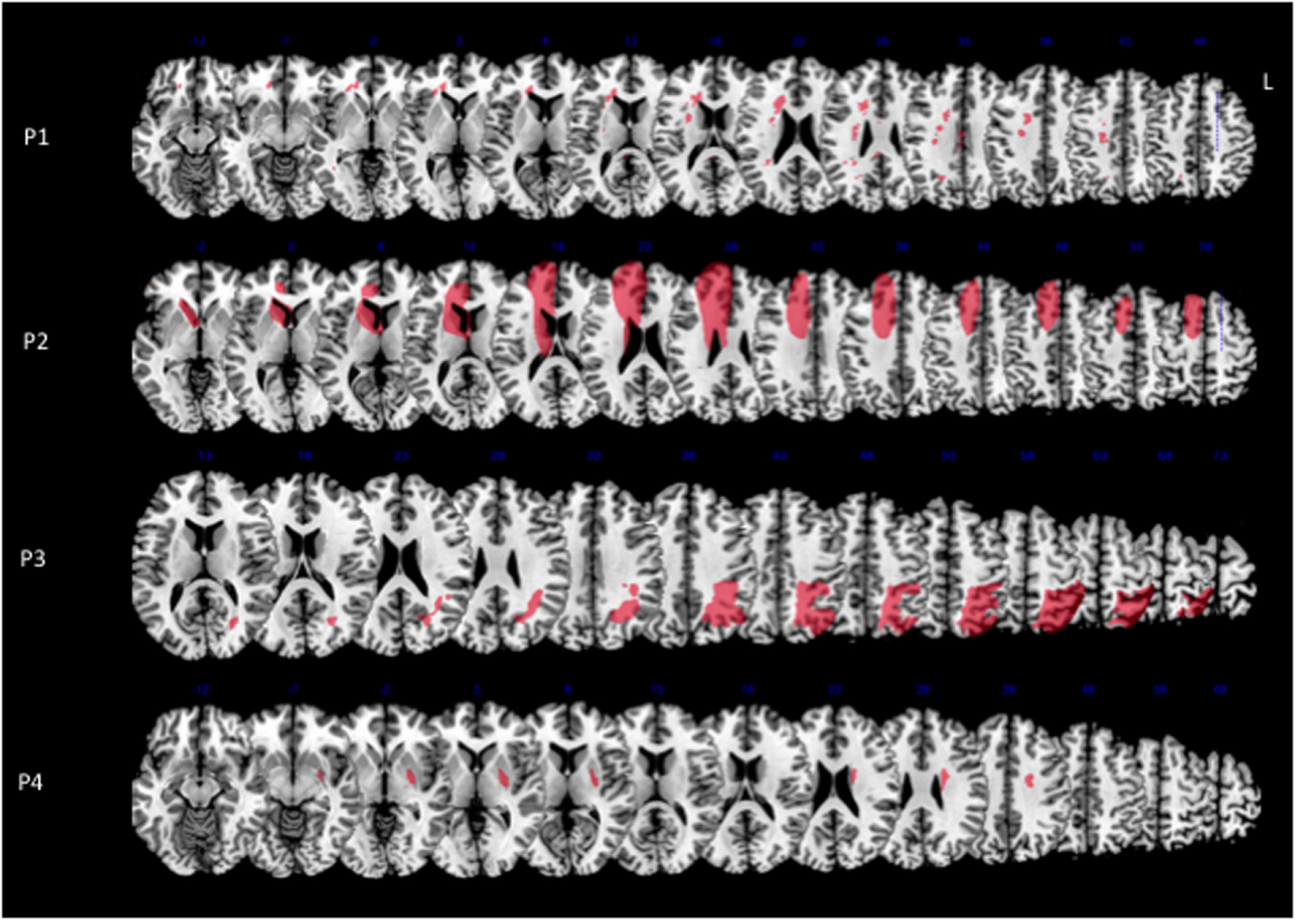
**Lesion reconstructions for each MN patient on the axial sections of the Montreal Neurological Institute (MNI) standard brain in radiological convention (L, left)**.

**Figure 2:**
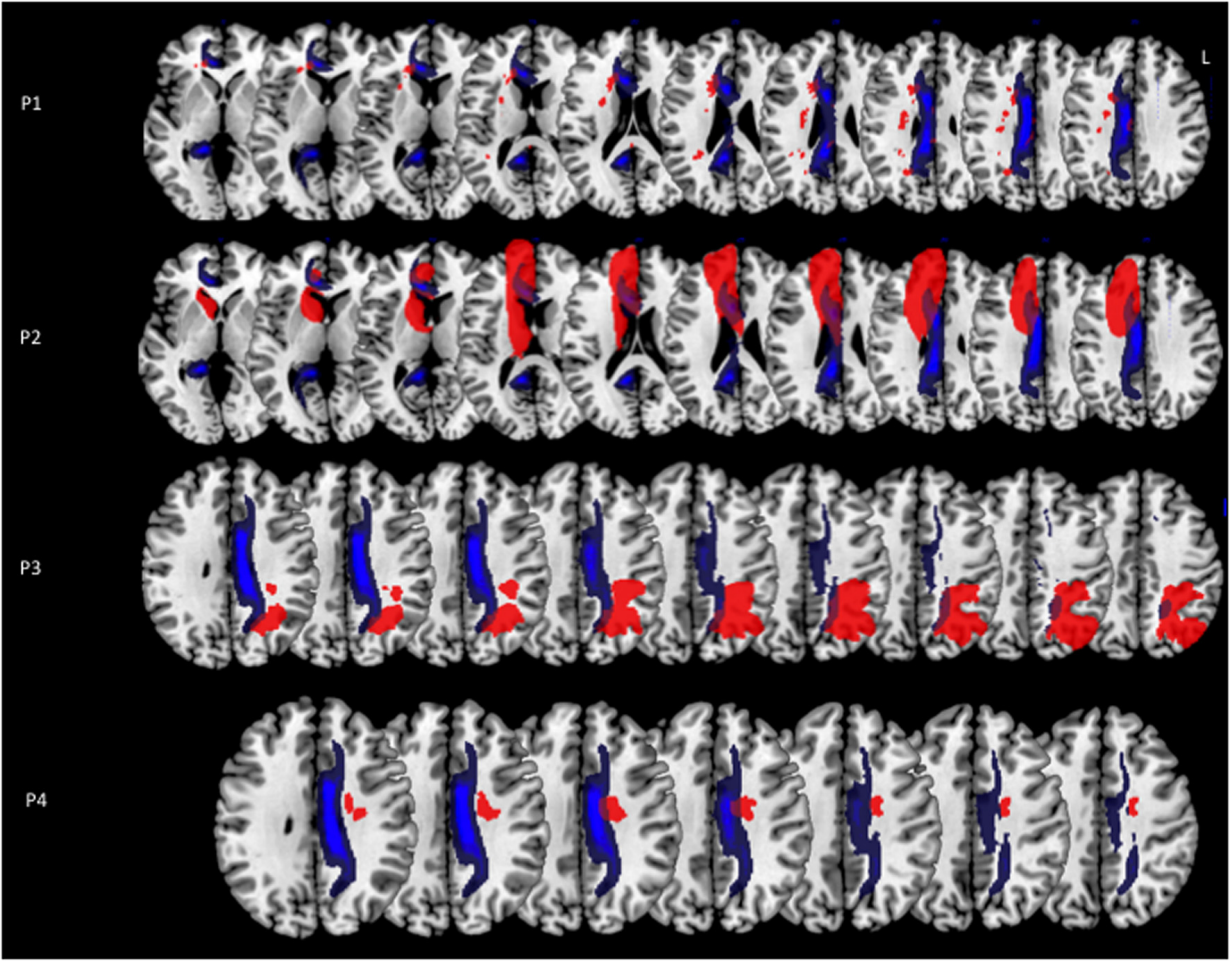
**Lesion reconstructions (in red) for each MN patient on the axial sections of the Montreal Neurological Institute (MNI) standard brain in radiological convention (L, left)**. A reconstruction of the cingulum (in blue) from a sample of 40 healthy subjects is superimposed to the images (for details see Thiebaut de Schotten et al., 2011b). Lesion and reference composite maps are displayed in order to show the maximal overlap in each patient.

We provide a clinical and anatomical description of the following chronic vascular patients: four patients presenting signs of MN, four patients showing signs of left visual neglect but no MN, and three patients with left hemiplegia and no MN. Among the four MN patients, two (patients 1 and 2) had left MN after a right hemisphere lesion; the two remaining patients (3 and 4) showed right MN after a left hemisphere lesion. Patient 4 (with left hemisphere lesion) was left-handed. MN was severe in patient 3, mild in the other patients. Three patients had mild signs of contralesional visual neglect, left-sided in patient 1, right-sided in patients 3 and 4. Figures 1, 2 and Table 1 summarize the clinical and anatomical results concerning MN patients.

Patient 2 had a large pre-Rolandic lesion involving right parasagittal frontal areas, including SMA and pre-SMA, as well as the cingulate cortex. In patient 3, who presented with the most severe MN, the maximal extent of the lesion was post-Rolandic, including the left parietal lobe, which was damaged in its dorsal and medial aspects. Patients 1 and 4 had smaller lesions. In patient 1, there were only diffuse white matter lesions in the corpus callosum and cingulum bundle. Patient 4 had right putaminal damage associated with diffuse white matter disease. To summarize, the distribution of gray matter involvement was highly heterogeneous, centered on frontal medial regions in patient 2, on parietal regions in patient 3, and on putamen in patient 4. There was no demonstrable gray matter involvement for patient 1.

Concerning the long-range white-matter fasciculi, the cingulum bundle was damaged in all the MN patients, at different levels in its rostro-caudal axis; the superior longitudinal fasciculus was lesioned in patients 1 and 3, with a possible involvement in patient 4 (Figure 2).

**Figure 3:**
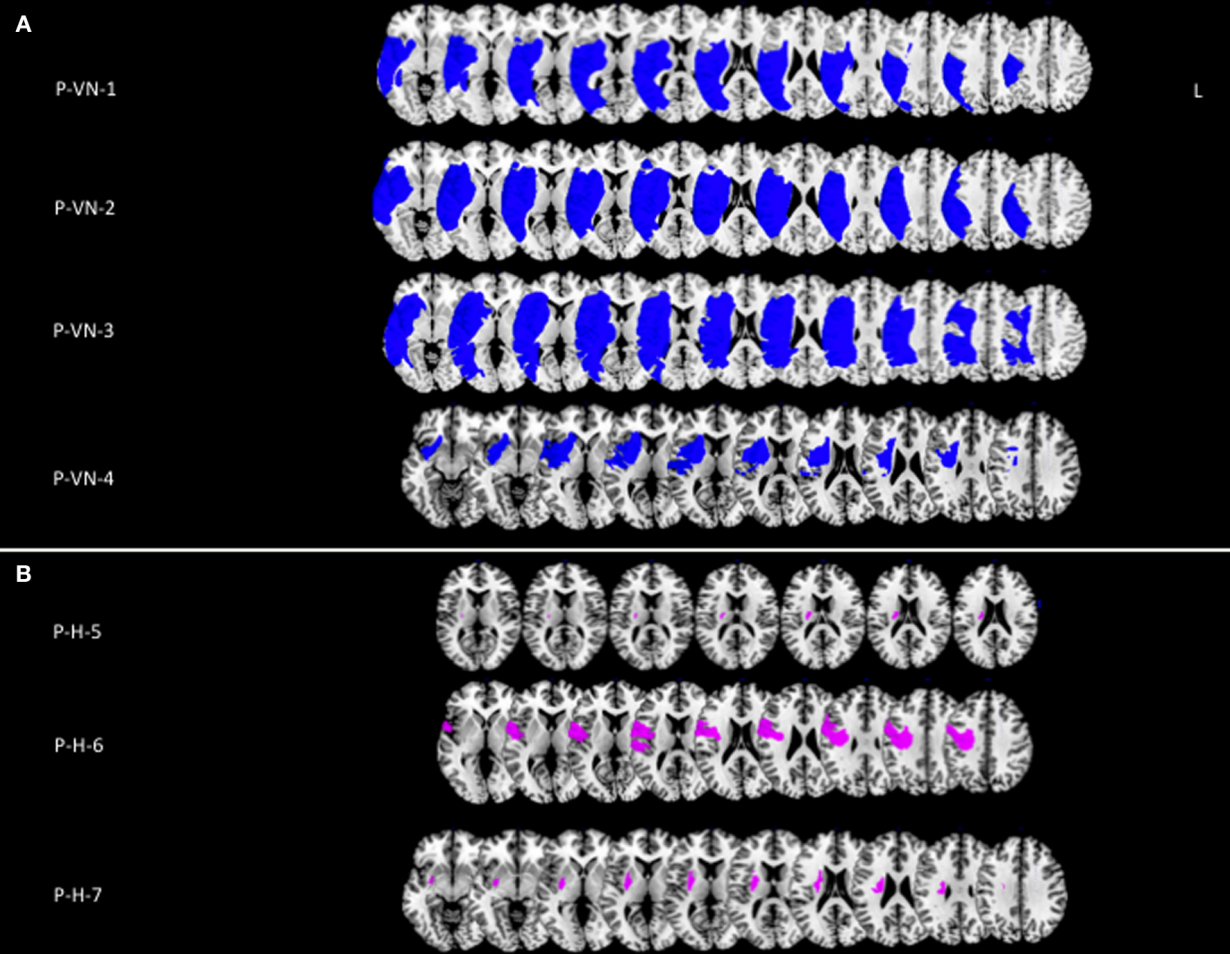
**(A)** Lesion reconstructions (in blue) for four patients with left visual neglect, without MN (P-VN). **(B)** Lesion reconstructions (in violet) for three patients with left hemiplegia, without signs of visual neglect or MN (P-H).

Three out of the four control patients with visual neglect showed large lateral brain lesions, involving parietal, temporal and frontal cortices, and the superior longitudinal fasciculus (Figure 3). In the remaining patient, the lesion was mainly anterior, involving lateral frontal and temporal cortices, and the superior longitudinal fasciculus. Control patients with hemiplegia had lesions of the internal capsule and the putamen (two cases), and of the lateral frontal rolandic operculum, precentral gyrus and superior lateral temporal cortices (one case). None of the control patients had damage involving the cingulum bundle (Figure 3).

Supplementary figure shows the lesion (largely including medial frontal and parietal regions) and the cingulum involvement in the excluded patient (Figure S1).

**Figure 4:**
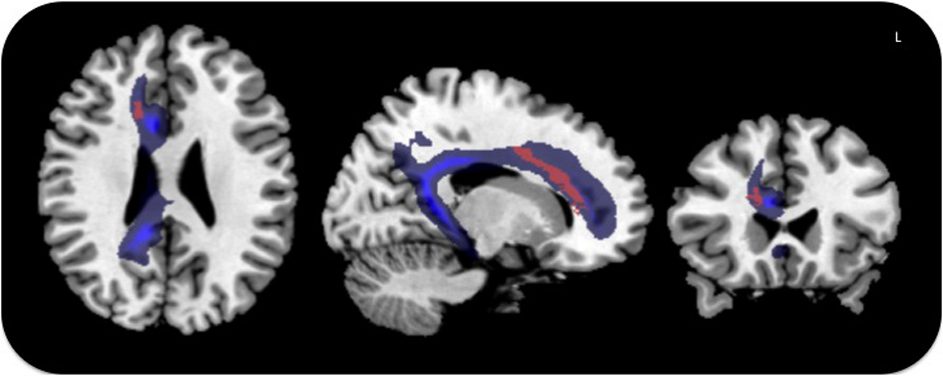
**Voxel-based lesion symptom mapping in MN patients *vs*. the control groups**. Lesion overlap contrast yielded maximal involvement of the anterior cingulum shown in red. The trajectory of the cingulum bundle (Thiebaut de Schotten et al., 2011b) is displayed in blue.

These results were corroborated by statistical comparison between lesion locations in MN patients and control groups (VN and H, without MN), which yielded maximal involvement of the anterior white matter cingulum bundle, centered on MNI coordinates *x* = 16, *y* = 26, *z* = 21 (Z-score = 2.13, *p* < 0.05, corrected for FDR) (Figure 4).

## Discussion

The present study aimed at exploring the pathophysiological and anatomical bases of MN, taking into account the proposed distinction between lateral and medial motor systems (Haggard, 2008). All our patients with MN had an involvement of the cingulum bundle, which was instead spared in all patients with visual neglect or hemiplegia, but no MN. The brain circuitry of voluntary action is still debated (Goldberg, 1985). Primary motor cortex (M1) is a final common station of several circuits important for voluntary action (Haggard, 2008). A lateral motor circuit originates from lateral parietal regions and carries information concerning sensory aspects of movement to the lateral premotor regions, which in turn transfer this information to M1. This network seems to be mainly implicated in stimulus-driven actions (e.g., object grasping and manipulation), and might arbitrate between a range of alternative actions, when an action is required (e.g., in forced choice). Another, more medial system seems instead important for the pure initiation of voluntary action, i.e., to decide the “whether,” “what,” and “when” of an action (Haggard, 2008). Regions in the medial system include the supplementary motor area (SMA), the pre-SMA, the cingulum, the lateral prefrontal cortex, and the basal ganglia. SMA and pre-SMA are functionally and structurally interconnected with the basal ganglia, lateral prefrontal cortex, cingulate cortex, as well as with the lateral and medial parietal lobe (precuneus) (Cavanna and Trimble, 2006). In MN, the selective impairment of spontaneous motility suggests a prevalent dysfunction of the medial motor system, but this relationship was hitherto undemonstrated, due to the insufficient level of detail of the previously available anatomical evidence.

In our four patients, MN was associated with vascular strokes in highly heterogeneous gray matter sites. The only consistent lesion across all the MN patients was damage to the cingulum bundle. The cingulum bundle is considered as a part of the limbic system. It runs medially in each hemisphere, just over the cingulate gyrus, with which it is connected along its entire course. Longer fibers directly connect medial temporal regions with sub-genual frontal areas. Shorter fibers connect adjacent areas in medial parietal and frontal lobes (Catani and Ffytche, 2005; Catani et al., 2013). Hence, the cingulum is a major pathway of the medial motor system, and a crucial connection between this system and limbic structures, which underlie motivational aspects of actions (Devinsky et al., 1995). Damage to the cingulum is thus likely to disrupt the integrated functioning of the medial motor system, with subsequent impaired SMA and pre-SMA activity and underutilization of the contralateral limbs. An imbalance between left and right medial systems may result in the impaired motor inhibition described in MN patients (Coulthard et al., 2008). Supporting evidence to this claim comes from a study (Laplane et al., 1977) in epileptic patients, who had severely decreased spontaneous movements of contralateral limbs after unilateral SMA corticectomy, and from the demonstration of impaired mesial frontal and putaminal activation in Parkinson's disease patients (Playford et al., 1992). Recently, Garbarini et al. (2013) reported the anatomical brain lesion sites of two patients with MN. Consistent with the present results, in one patient (MN-7) damage of rostral cingulum is clearly identified. The other patient (MN-8), whose brain injury was similar to that sustained by the present patient 3, had probable damage to more caudal aspects of the cingulum.

The connectivity of the cingulum bundle suggests its likely critical role as an interface between limbic structures (Catani et al., 2013), where motivation to act originates, and the neocortical mantle which elaborates motivation into a fully formed specification of the overt act (Brown, 1977). Thus, a lesion involving the cingulum bundle could disrupt the flow of information from limbic structures to neocortical regions and lead to MN. Studies in monkeys (Schmahmann and Pandya, 2006) and humans (Thiebaut de Schotten et al., 2011a) suggest that just dorsal to the cingulum runs a fiber bundle (SLF I), which links, among other areas, the medial parietal and frontal regions. Disconnection of SLF I could thus also be implicated in the pathogenesis of MN.

The clinical impact of motor neglect is indisputable. In a study on 52 stroke patients (Siekierka-Kleiser et al., 2006), 19 developed MN, which was persistent in 14 of these cases. These MN patients were more severely compromised in their daily lives than those who did not suffer from MN. Diagnosis of MN is important, also because some intervention is possible. MN can be improved by forced-use therapy (Liepert et al., 1998; Van Der Lee et al., 1999), caloric vestibular stimulation (Rode et al., 1998) or optokinetic stimulation (Vallar et al., 1997). Heidler-Gary et al., 2013 asked 93 non-hemiplegic patients with acute (<48 h) right hemisphere stroke to press a hand-held counter with the right hand alone, left hand alone, or the two hands simultaneously. For 20 patients, left-hand clicking decreased when the right hand had to move at the same time (motor extinction). Voxel-based analysis highlighted damage in the white matter lateral to the thalamus, in the right subcortical temporal cortex. However, it is not clear whether motor extinction for movements performed upon external instructions can be considered homogeneous to MN, which is characterized by decreased spontaneous movements.

In addition to MN, three of the present patients also demonstrated signs of contralesional visual neglect. All these patients had damage to the lateral fronto-parietal networks connected by the superior longitudinal fasciculus, consistent with abundant previous evidence linking crucial aspects of the neglect syndrome to lateral fronto-parietal dysfunction (Bartolomeo et al., 2007; Doricchi et al., 2008; Corbetta and Shulman, 2011). Importantly, the only patient who did not show signs of visual neglect had an extensive frontal parasagittal lesion, and no detectable lesion of the superior longitudinal fasciculus. The dissociation of MN from visual neglect is described in the literature (Laplane and Degos, 1983) and was present in one of our MN cases. These occurrences logically exclude that MN and visual neglect always result from the same underlying impairment, as further demonstrated by the present anatomical analysis. However, visual or personal neglect might well contribute to the presence and severity of MN in some cases, by impairing patients' ability to direct their attention toward their contralesional limbs.

To conclude, models of neural correlates of spontaneous motor acts are based on a distribution of functional roles among cortical areas (Goldberg, 1985; Passingham, 1987; Haggard, 2008). The present multiple single-case study suggests that the cingulum is a key connection pathway in these networks, linking (i) posterior parietal areas, important for spatial attention and sensory-motor input integration, with (ii) frontal medial motor areas, involved in motor planning and execution, (iii) medial limbic structures, involved in motivation to action, and (iv) subcortical structures involved in motor control. The case of patient 1, with damage restricted to the white matter, suggests that at least in some cases an isolated cingulum disconnection can result in MN.

The low number of explored patients in the present study results from the relative rarity of the syndrome and its tendency to spontaneous recovery, as well as from our choice to include only chronic patients without primary motor deficits. Nevertheless, the present anatomical evidence strongly suggests an important role for white matter damage to the cingulum, independent of the lesion location in the rostro-caudal brain axis or of its hemispheric side. Our results suggest that cingulum damage is central in the unilateral dysfunction of a medial motor system resulting in defective spontaneous motility of the contralateral limbs after brain damage.

## Author contributions

Raffaella Migliaccio and Paolo Bartolomeo: conception of the work, acquisition, analysis, and interpretation of data for the work; drafting the work. Florence Bouhali and Federica Rastelli: acquisition, analysis, and interpretation of data and revising the work critically for important intellectual content. Pascale Pradat-Diehl, Sophie Ferrieux, Celine Arbizu, and Stephane Vincent: acquisition of data, and revising the manuscript. All authors approved the final version to be published; and agreed to be accountable for all aspects of the work in ensuring that questions related to the accuracy or integrity of any part of the work are appropriately investigated and resolved.

### Conflict of interest statement

The authors declare that the research was conducted in the absence of any commercial or financial relationships that could be construed as a potential conflict of interest.

## Supplementary material

Figure S1 **Lesion reconstructions (in red) for MN patient excluded from the study because of severe frontal syndrome**. The lesion is displayed on the axial sections of the Montreal Neurological Institute (MNI) standard brain in radiological convention (L, left). A reconstruction of the cingulum (in blue) from a sample of 40 healthy subjects is superimposed to the lesion (for details see Thiebaut de Schotten et al., 2011b).
