# Supplementary figures and images for: Damage to the medial motor system in stroke patients with motor neglect
Source: Front Hum Neurosci. 2014 Jun 11;8:408. doi: 10.3389/fnhum.2014.00408 (PMC4052665; doi:10.3389/fnhum.2014.00408)

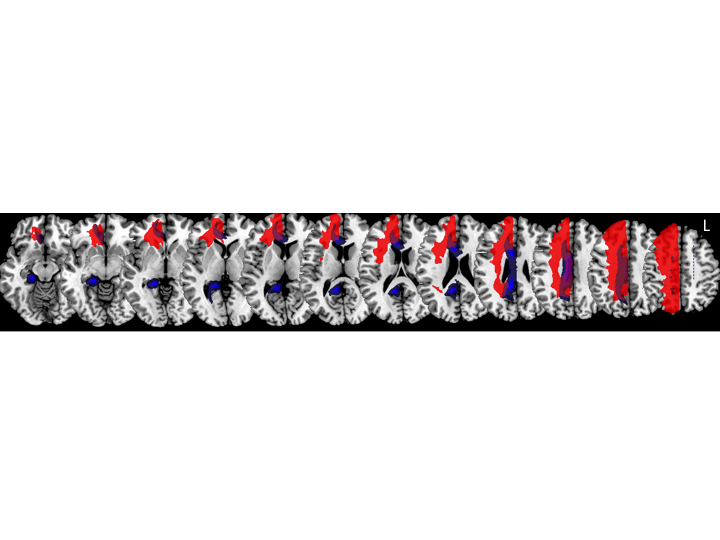

Supplement: Figure S1 — Lesion reconstructions (in red) for MN patient excluded from the study because of severe frontal syndrome. The lesion is displayed on the axial sections of the Montreal Neurological Institute (MNI) standard brain in radiological convention (L, left). A reconstruction of the cingulum (in blue) from a sample of 40 healthy subjects is superimposed to the lesion (for details see Thiebaut de Schotten et al., 2011b). [file Presentation1.ZIP › Migliaccio_Supplementary Figure_1.TIFF]
