# Supplementary material for: Damage to the medial motor system in stroke patients with motor neglect
Source: Front Hum Neurosci. 2014 Jun 11;8:408. doi: 10.3389/fnhum.2014.00408 (PMC4052665; doi:10.3389/fnhum.2014.00408)
Supplement: Supplementary file 2 [file DataSheet1.DOCX]

**Damage to the medial motor system in stroke patients with motor neglect**

Migliaccio R,* ^1,2,3^ Bouhali F,^1^ Rastelli F,^1,4^ Ferrieux S,^2^ Arbizu C,^2^ Vincent S,^4^ Pradat-Diehl P,^4,5^ and Bartolomeo P^1,2,3^.

^1^ Inserm UMR-S 1127; UPMC-Paris 6; CNRS UMR 7225, Brain and Spine Institute, Groupe Hospitalier Pitié-Salpêtrière, Paris, France.

^2^ AP-HP, Groupe Hospitalier Pitié-Salpêtrière, Fédération de Neurologie, IM2A, Paris, France.

^3^ Department of Psychology, Catholic University, Milan, Italy.

^4^ AP-HP, Service de Médecine Physique et Réadaptation, Groupe Hospitalier Pitié-Salpêtrière, Paris, France. ^5^ ER 06, UPMC, Service de MPR, Groupe Hospitalier Pitié-Salpêtrière, Paris, France.

**Supplementary table.**

# Motor Neglect assessment

**1) Clinical assessment**

| ***Patient’s information***  **Name:**  **Gender:**  **Age:**  **Affected Side: left/right**  **Hemiplegia: yes/no** | **Visual neglect: yes/no**  **Handedness:**  **Date of assessment:**  **Examiner:**  **Disease onset:** |
| --- | --- |

| **Muscular strength:** |  |
| --- | --- |
| Proximal strength: | Distal strength: |

| **Walking:** |  |  |  |
| --- | --- | --- | --- |
| Arm swing | Present | Reduced | Absent |
|  | 0 | 1 | 2 |
| Positioning of the affected leg | Normal | Slightly abnormal | Very abnormal (e.g., affected leg lagging behind) |
|  | 0 | 1 | 2 |
| **Sitting:** |  |  |  |
| Positioning of the affected hand when sitting | Normal | Slightly abnormal (neglected hand) | Very abnormal |
|  | 0 | 0.5 | 1 |
| Positioning of the affected leg when sitting | Normal | Slightly uncomfortable | Very abnormal  (e.g., leg beside the chair) |
|  | 0 | 0.5 | 1 |
| **General posture** | Symmetrical position | Slight asymmetry  (Posture is slowly corrected after solicitation) | Important asymmetry  (Posture is not corrected after solicitation) |
|  | 0 | 1 | 2 |

| **Hypometria** | Normal  (complete movement) | Slight hypometria | Severe hypometria |
| --- | --- | --- | --- |
| Finger-nose movement  (at first with eyes open, and then closed) | 0 | 1 | 2 |
| Reach for an object with the affected hand, then with the unaffected hand  (with free gaze, then when fixing examiner) | 0 | 1 | 2 |
| **Difficulties in initiating these latter movements** | None | Slight | Severe |
|  | 0 | 1 | 2 |
| **Bimanual tasks:** | Success at all tasks  0 | Success at 2 or 3 tasks  1 | Success at only 1 task or none  2 |
| - Clapping hands |  |  |  |
| - Opening and closing a bottle |  |  |  |
| Buttoning coat |  |  |  |
| Maintaining the paper leaf when writing |  |  |  |
| Folding a paper in 4 and putting it into an envelope |  |  |  |
| **Central reception of a tennis ball** | Normal | Clumsy | Impossible |
|  | 0 | 1 | 2 |
| **Gesturing**  Does the patient accompany his/her talking with affected hand gesturing – comparing to the right hand? (when asked to tell his/her clinical history for instance) | Yes | A little bit | Not at all |
|  | 0 | 1 | 2 |

**2) Evaluation Form to be completed by rehabilitation professional staff**

**Patient’s name:**

**Therapist name:**

**Date:**

**Affected side: left/right**

Have you ever noticed that the patient…

|  | No | Yes, a bit | Yes, a lot |
| --- | --- | --- | --- |
| Under-uses his/her affected hand in situations which would normally require its use? | 0 | 2 | 4 |
| Adopts asymmetrical postures in which his/her affected (upper and/or lower) limbs are in seemingly uncomfortable positions? | 0 | 2 | 4 |
| Demonstrates a lack of natural arm swing when walking? | 0 | 2 | 4 |
| Perform movements of reduced amplitude with his/her affected limbs (especially with the upper limb)? | 0 | 2 | 4 |

**3) Ecological task (tea preparation)**

*Case of a simple interaction: The dominant hand is not the affected one.*

The patient is placed in front of a table on which lay a number of objects required to prepare the tea: a teapot filled with hot water, an individually wrapped tea bag, a sugar stick and a tea spoon. He/she is asked to prepare tea as he/she would do at home.

|  | **Used hand** | | | | | |
| --- | --- | --- | --- | --- | --- | --- |
| **Kettle or teapot** |  | | |  | | |
| Kettle / Teapot handle grasp | Unimpaired dominant hand | | | Impaired non-dominant hand | | |
|  | 0 | | | -4 | | |
| Mug or teapot holding with the other hand when pouring water | Present | Poor | Absent | Present | Poor | Absent |
|  | 0 | 2 | 4 | 0 | -2 | -4 |
| **Tea bag** |  | | |  | | |
| Grasping | Unimpaired dominant hand | | | Impaired non-dominant hand | | |
|  | 0 | | | -1 | | |
| Fine-grained motor acts for tea bag opening | Unimpaired dominant hand | | | Impaired non-dominant hand | | |
|  | 0 | | | -1 | | |
| Tea bag maintaining with the other hand | Present | Poor | Absent | Present | Poor | Absent |
|  | 0 | 2 | 4 | 0 | -2 | -4 |
| **Putting sugar** |  | | |  | | |
| Tea spoon grasping | Unimpaired dominant hand | | | Impaired non-dominant hand | | |
|  | 0 | | | -2 | | |
| Handle holding during when mixing | Present | Poor | Absent | Present | Poor | Absent |
|  | 0 | 1 | 2 | 0 | -1 | -2 |
| **Grasping of the mug handle** | Unimpaired dominant hand | | | Impaired non-dominant hand | | |
|  | 0 | | | -4 | | |

*Case of a complex interaction: The dominant hand is the affected one.*

The patient is placed in front of a table on which lay a number of objects required to prepare the tea: a teapot filled with hot water, an individually wrapped tea bag, a sugar stick and a tea spoon. He/she is asked to prepare tea as he/she would do at home.

|  | **Used hand** | | | | | |
| --- | --- | --- | --- | --- | --- | --- |
| **Kettle or teapot** |  | | |  | | |
| Kettle / Teapot handle grasp | Impaired dominant hand | | | Unimpaired non-dominant hand | | |
|  | -2 | | | 6 | | |
| Mug or teapot holding with the other hand when pouring water | Present | Poor | Absent | Present | Poor | Absent |
|  | 0 | -2 | -4 | 0 | 2 | 4 |
| **Tea bag** |  | | |  | | |
| Grasping | Impaired dominant hand | | | Unimpaired non-dominant hand | | |
|  | -1 | | | 3 | | |
| Fine-grained motor acts for tea bag opening | Impaired dominant hand | | | Unimpaired non-dominant hand | | |
|  | -1 | | | 3 | | |
| Tea bag maintaining with the other hand | Present | Poor | Absent | Present | Poor | Absent |
|  | 0 | -2 | -4 | 0 | 2 | 4 |
| **Putting sugar** |  | | |  | | |
| Tea spoon grasping | Impaired dominant hand | | | Unimpaired non-dominant hand | | |
|  | -1 | | | 3 | | |
| Handle holding during when mixing | Present | Poor | Absent | Present | Poor | Absent |
|  | 0 | -1 | -2 | 0 | 1 | 2 |
| **Grasping of the mug handle** | Impaired dominant hand | | | Unimpaired non-dominant hand | | |
|  | -2 | | | 6 | | |
